# Dual interleukin-17A/F deficiency protects against acute and chronic response to cigarette smoke exposure in mice

**DOI:** 10.1038/s41598-021-90853-9

**Published:** 2021-06-01

**Authors:** Hiroo Wada, Masuo Nakamura, Shin-Ichi Inoue, Akihiko Kudo, Tomoko Hanawa, Yoichiro Iwakura, Fumie Kobayashi, Hiroshi Kamma, Shigeru Kamiya, Kazuhiro Ito, Peter J. Barnes, Hajime Takizawa

**Affiliations:** 1 Department of Public Health, Graduate School of Medicine, Juntendo University, 2-1-1 Hongo, Bunkyo, Tokyo, 113-8421 Japan; 2 Department of Respiratory Medicine, Kyorin University School of Medicine, 6-20-2 Shinkawa, Mitaka, Tokyo 181-8611 Japan; 3 Division of Immunology, Department of Molecular Microbiology and Immunology, Graduate School of Biomedical Sciences, Nagasaki University, 1-12-4, Sakamoto, Nagasaki, Nagasaki 852-8523 Japan; 4 Department of Microscopic Anatomy, Kyorin University School of Medicine, Shinkawa, Mitaka, Tokyo 181-8611 Japan; 5 Department of Infectious Diseases, Kyorin University School of Medicine, Shinkawa, Mitaka, Tokyo 181-8611 Japan; 6 Division of Animal Immunology, Research Institute for Biological Sciences, Tokyo University of Science, 2669 Yamazaki, Noda, Chiba Japan; 7 Department of Environmental Science, School of Life and Environmental Science, Azabu University, Chuo-ku, Sagamihara, Kanagawa 252-5201 Japan; 8 Department of Pathology, Kyorin University School of Medicine, Shinkawa, Mitaka, Tokyo 181-8611 Japan; 9 Airway Disease Section, National Heart and Lung Institute, Imperial College London, London, UK; 10 Present Address: Nakamura Clinic, 2-44-15 Kami-ishiwara, Chofu, Tokyo Japan

**Keywords:** Cell biology, Environmental sciences, Diseases, Medical research, Pathogenesis

## Abstract

IL-17A and IL-17F are both involved in the pathogenesis of neutrophilic inflammation observed in COPD and severe asthma. To explore this, mice deficient in both *Il17a* and *Il17f* and wild type (WT) mice were exposed to cigarette smoke or environmental air for 5 to 28 days and changes in inflammatory cells in bronchoalveolar lavage (BAL) fluid were determined. We also measured the mRNA expression of keratinocyte derived chemokine (*Kc*), macrophage inflammatory protein-2 (*Mip2)*, granulocyte–macrophage colony stimulating factor (*Gmcsf)* and matrix metalloproteinase-9 (*Mmp9* ) in lung tissue after 8 days, and lung morphometric changes after 24 weeks of exposure to cigarette smoke compared to air-exposed control animals. Macrophage counts in BAL fluid initially peaked at day 8 and again on day 28, while neutrophil counts peaked between day 8 and 12 in WT mice. Mice dual deficient with *Il17a and 1l17f* showed similar kinetics with macrophages and neutrophils, but cell numbers at day 8 and mRNA expression of *Kc*, *Gmcsf* and *Mmp9* were significantly reduced. Furthermore, airspaces in WT mice became larger after cigarette smoke exposure for 24 weeks, whereas this was not seen dual *Il17a and 1l17f* deficient mice. Combined *Il17a* and *Il17f* deficiency resulted in significant attenuation of neutrophilic inflammatory response and protection against structural lung changes after long term cigarette smoke exposure compared with WT mice. Dual IL-17A/F signalling plays an important role in pro-inflammatory responses associated with histological changes induced by cigarette smoke exposure.

## Introduction

The IL-17 family of cytokines has six members, of which IL-17A and IL-17F are highly homologous and form a homodimer or a heterodimer and bind to a complex receptor of IL-17RA and IL-17RC, so that they share similar biological effects^1–3^ and appear to work in an overlapping way^3,4^. Several studies suggest that IL-17A and/or IL-17F are associated with airway inflammatory diseases, such as severe asthma and COPD^5,6^. Alveolar macrophages from COPD patients and healthy subjects express both IL-17A and IL-17F^7^ and cells positively immunostained for IL-17A are increased in the airway submucosa in patients with severe asthma and COPD^8^. Sputum IL-17A as well as CXCL8 levels are also reported to be higher in more severe COPD patients, and are inversely related to airway obstruction^9^. In patients with COPD, sputum levels of IL-17A are increased during exacerbations, and come down again after recovery, whereas sputum IL-17F levels show lesser increases^10^. Likewise, IL-17A and IL-17F are increased in asthma patients, with the levels higher in more severe disease and associated with increased sputum neutrophils^11^. These data suggest that IL-17A and IL-17F are both involved in the pathogenesis of neutrophilic inflammation observed in COPD patients and some with severe asthma.

The role of IL-17 in airway inflammations in COPD has been investigated using cigarette smoke exposure in mice. Treatment with a neutralizing antibody against IL-17A partially restored neutrophil recruitment to the lung induced by cigarette smoke exposure for 10 weeks^12^. Cigarette smoke exposure for 4 weeks resulted in the recruitment of neutrophils to the lung, and the enhanced expression of IL-17A, but not IL-17F. Neutrophil recruitment to the lung after cigarette smoke exposure was reduced in IL-17A gene-deficient mice^13,14^, although protection against the increase in alveolar size was not observed^2,14^. In another COPD mouse model induced by intratracheal elastase, there was an increase in lung *Il17a* expression and neutrophil recruitment to the lung, as well as alveolar airspace enlargement. Both neutrophil recruitment to the lung and airspace enlargement in murine model of elastase-induced emphysema were again only partially attenuated in *Il17a* deficient mice^15^. Infectious sinusitis is reported to occur exclusively in mice deficient both in *Il17a* and *Il17f* genes, while mice deficient in either *Il17a* or *Il17f* did not show the same phenotype^4^. In addition, another study reported that IL-17F expression was increased in *Il17a* -deficient mice, while IL-17A treatment suppresses *Il17f* expression, all suggesting that these related cytokines may overlap their functions and why double *il17a/f* knock-out mice are more informative^16^. Therefore, to explore in both acute neutrophilic inflammation and its chronic consequences, the present study investigated *Il17a/f* double-knockout mice exposed to cigarette smoke for over 28 days to study the inflammatory response and then for 24 weeks to study structural changes in peripheral lung .

## Results

### Time course of inflammatory response to inhaled cigarette smoke

**Figure 1:**
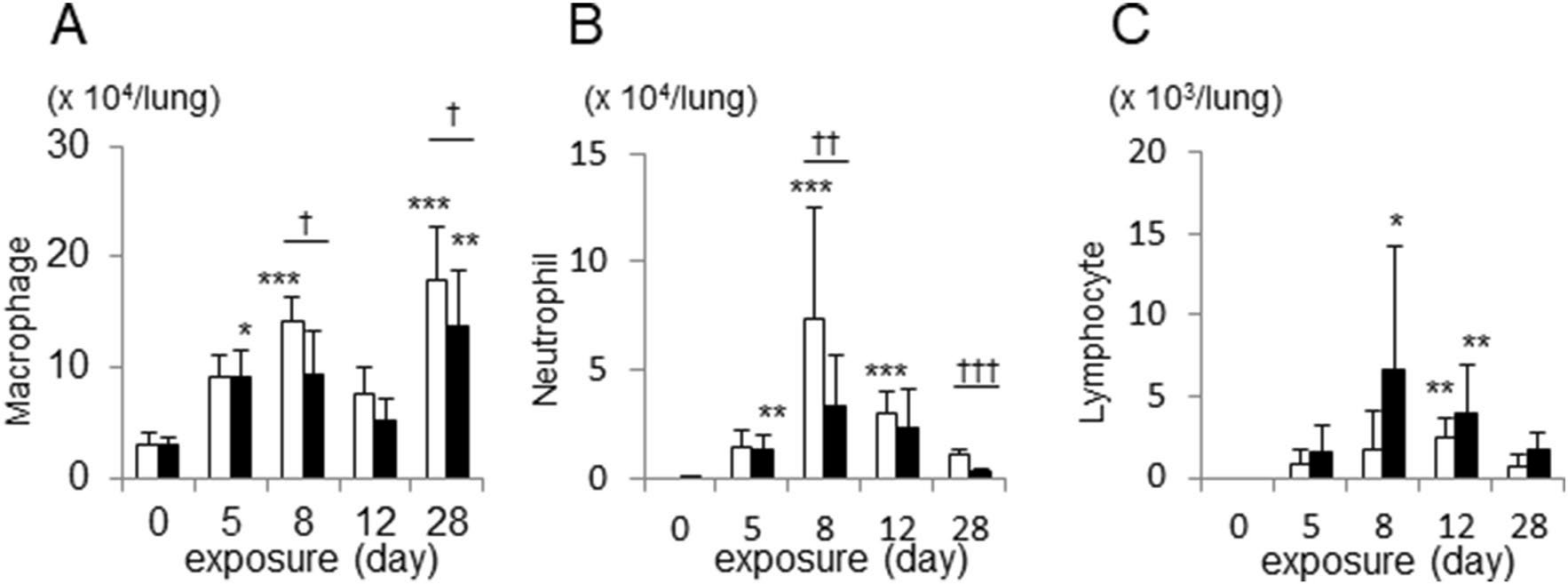
Time course of inflammatory cell counts in interleukin-17A/F (IL-17A/F) deficient mice compared to wild-type mice, following exposure to cigarette smoke over 28 days. Macrophages (**A**), neutrophils (**B**) and lymphocyte (**C**) in bronchoalveolar lavage (BAL) fluid are shown. Values are means ± SD of minimum of 6 animals per group (*p < 0.05, **p < 0.01, ***p < 0.001 compared with the group of day 0; ^†^p < 0.05, ^††^p < 0.01, ^†††^p < 0.001 compared between wild-type and Il-17A/F deficient mice on the same conditions of exposure).

BAL fluid macrophage counts in WT mice peaked at day 8 of cigarette exposure, and then declined to day 12 prior to another gradual increase up to day 28. *Il17a* and *Il17f* dual deficient mice showed similar kinetics although macrophage counts were significantly lower at day 8 and day 28 (p < 0.05, Fig. 1A). On the other hand, neutrophil counts peaked between day 8 and 12 in both groups of animals, followed by a decline towards the day 28. Neutrophil counts in *Il17a* and *Il17f* dual deficient mice were significantly lower both at day 8 and day 28 (p = 0.025 and p < 0.001 respectively) (Fig. 1B). Lymphocyte counts peaked at day 12 in the WT mice and the *Il17a* and *Il17f* dual -deficient mice showed a trend of increase in lymphocyte counts, but this was not statistically significant (Fig. 1C).

### Inflammatory response to cigrette smoke in IL-17-deficient mice at day 8

**Figure 2:**
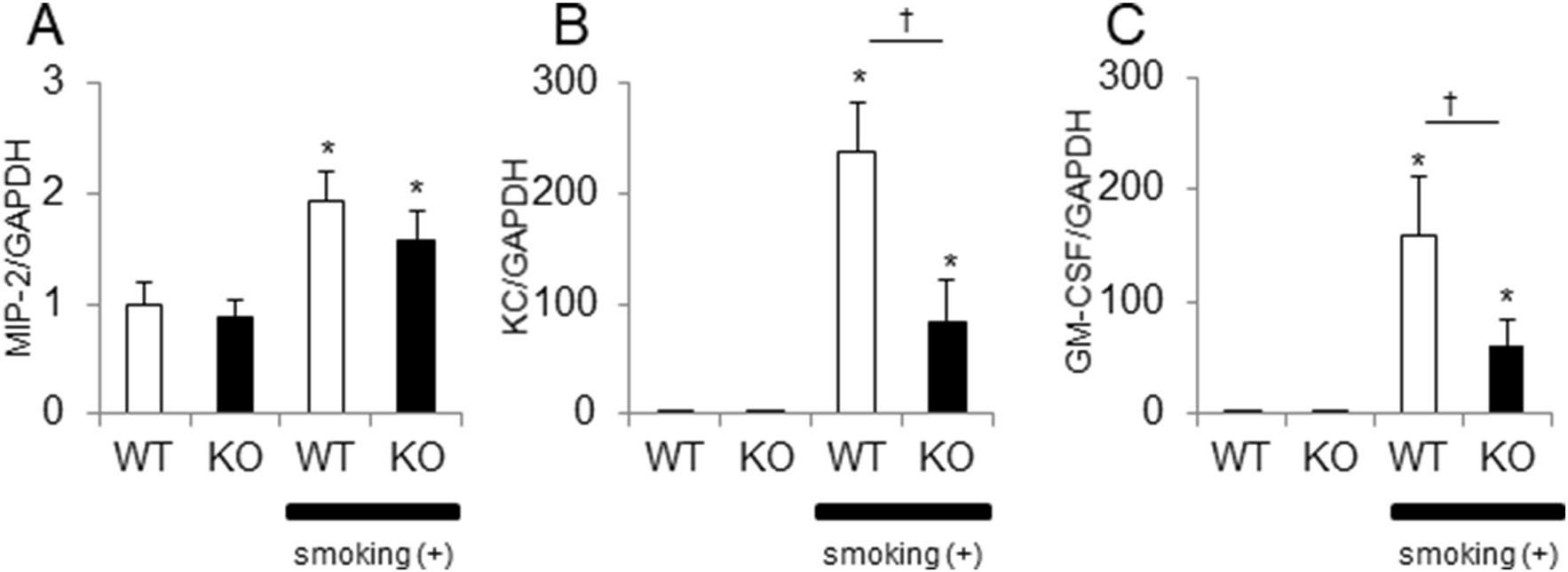
Inflammatory response induced by cigarette smoke exposure in *Il17a* and *Il17f* dual deficient mice compared to wild-type mice. Lung tissue mRNA expression of *Mip2* (**A**), *Kc* (**B**), and *Gmcsf* (**C**). Closed bars represent IL-17A/F deficient mice; open bars represent wild-type mice. Values are means ± SD of 4 to 8 animals per group (*p < 0.05, compared with air control group in the same genotype; ^†^p < 0.05 compared between *Il17a* and *Il17f* dual deficient and wild-type mice).

The mRNA levels of MIP-2 on day 8 were significantly increased in *Il17a* and *Il17f* dual deficient mice as well as in the WT mice, with no significant difference (Fig. 2A). *Kc* and *Gmcsf* expression was induced by cigarette smoke exposure in both genotypes, but the levels were significantly reduced in *Il17a* and *Il17f* dual deficient mice compared to control animals (Fig. 2B,C). The time course of the cellular response, as well as enhancement of mediator expression, after smoke exposure is consistent with previous reports^17–20^.

### Airspace enlargement and neutrophilic infiltrate

**Figure 3:**
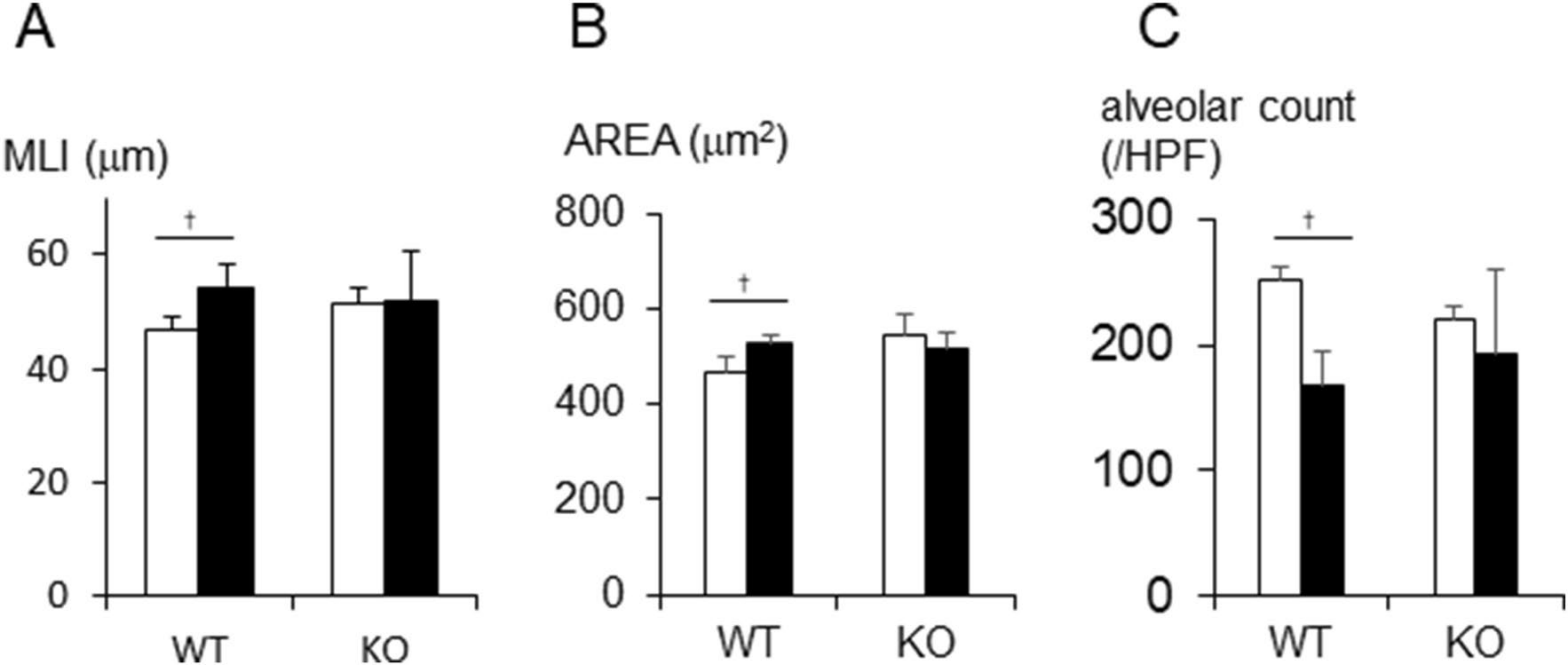
Airspace enlargement after cigrette smoke exposure for 24 weeks were compared between IL-17A/F deficient mice and wild type mice by determining mean linear intercepts (MLI) (**A**), area of average airspace area (**B**), and the number of alveoli in an unit area (**C**) on histologic samples of haematoxylin–eosin (HE) staining (low magnification). Closed bars represent *Il17a* and *Il17f* dual deficient mice; open bars represent wild-type mice. Values are means ± SD (n = 4 to 8 per group, †p < 0.05 compared with wild-type animals on environmental exposure).

**Figure 4:**
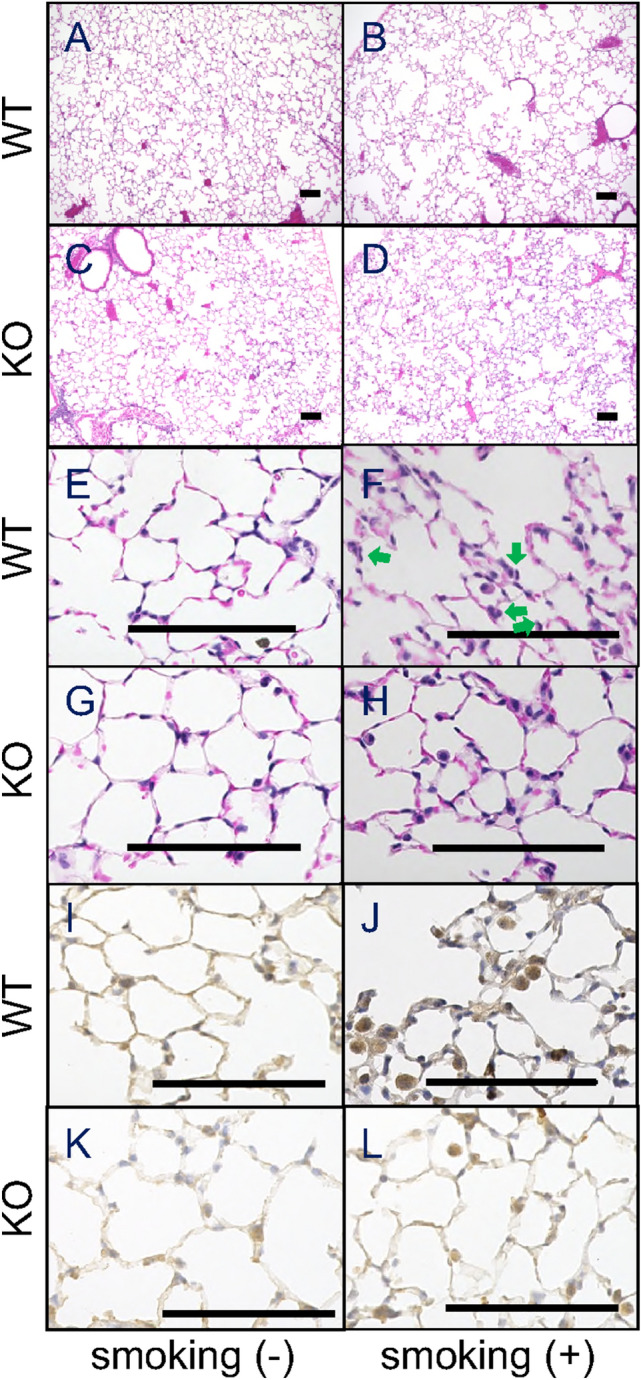
Representative histology with haematoxylin and eosin staining (low magnification) after exposure for 24 weeks, showing the comparison in airspace enlargement between wild-type mice on air (control, **A**), wild-type mice on cigarette smoke exposure (**B**), dual deficient mice on air (**C**), dual deficient mice on cigarette smoke exposure (**D**). Representative histology with haematoxylin and eosin staining (high magnification) as well as immuno-histochemistry (high magnification) against MMP-9 were shown in wild type mice on air (**E**,**I**) and those on cigarette smoke exposure (**F**,**J**) in comparison with dual deficient mice on air (**G**,**K**), and those after cigarette smoke exposure (**H**,**L**), respectively. It was shown that neutrophils were strongly positive in (**J**). Scale bar represents 100 micro-meter. Arrows indicate neutrophils.

Histological analysis of the lungs revealed a neutrophilic infiltrate and airspace enlargement after cigarette exposure. In WT mice, the mean linear intercept (MLI) (Fig. 3A) and the alveolar space area (Fig. 3B) were significantly increased after 24 weeks of cigarette smoke-exposure, while alveoli per unit area decreased (shown in Figs. 3C, 4A,B). In contrast, there was no alveolar changes in *Il17a* and *Il17f* dual deficient animals (Figs. 3A–C, 4C,D).

### MMP-9 positive cells

**Figure 5:**
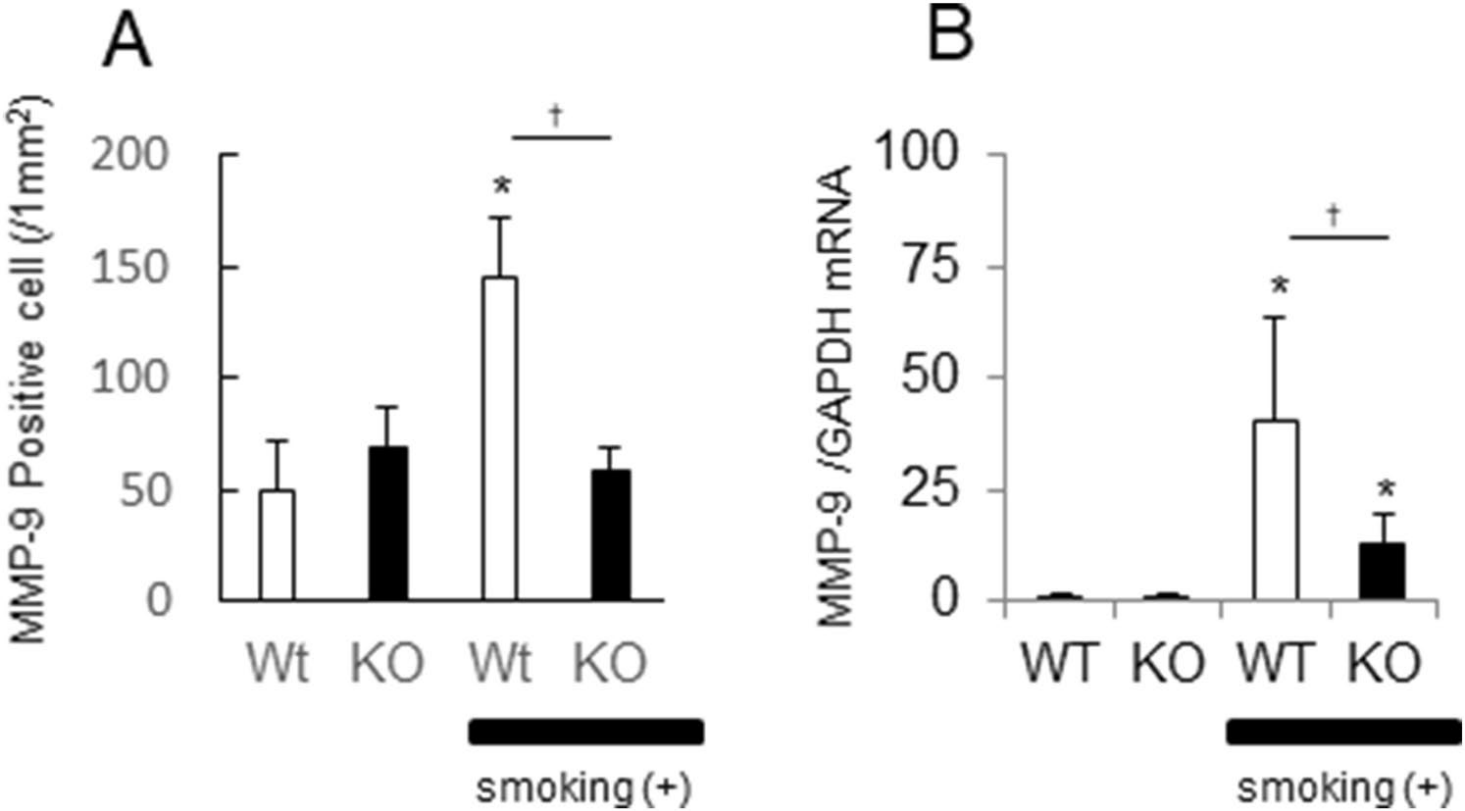
After 8 days of exposure, neutrophil recruitment was determined by the cell counts with MMP-9 immunostaining (**A**) and by the increased MMP-9 mRNA (**B**). Values are means ± SD of 4 to 8 animals per group (*p < 0.05 compared with the control animals on environmental exposure, and ^†^p < 0.05 compared between *Il17a* and *Il17f* dual deficient mice and wild-type mice).

Neutrophils were recruited to the alveoli in wild-type mice exposed to cigarette smoke for 8 days (Fig. 4E,F), but not in *Il17a* and *Il17f* dual deficient animals (Fig. 4G,H). MMP-9 positive cells identified by immunostaining were increased in the alveolar walls of WT mice exposed to cigarette smoke (Fig. 4J vs I; K vs L), but this was significantly blunted (p < 0.05) in *Il17a* and *Il17f* dual deficient animals (Fig. 5A). This was confirmed by measuring *Mmp9* mRNA levels, demonstrating that cigarette smoke exposure induced *Mmp9* expression in both groups but that with lower expression in *Il17a* and *Il17f* dual deficient mice (Fig. 5B).

## Discussion

The present study has demonstrated that *Il17a* and *Il17f* dual deficiency had a marked suppressive effect on macrophage and neutrophil recruitment to the lung after cigarette smoke exposure. Furthermore, *Il17a* and *Il17f* dual deficient mice were protected against enlargement of airspaces (emphysema) due to long-term cigarette exposure.

Previous studies showed that IL-17A in mice was a major mediator in short-term (2–10 weeks) cigarette smoke-induced airway inflammation, and in acute emphysema models using elastase^12–15^, whereas another study showed that neither IL-17A nor MMP-9 increased in expression after the elastase instillation in mice^21^. In addition, *Il17a* deficiency alone partially prevented the inflammatory responses^12,13,15^, and airspace enlargement was not protected (14% increase in mean chord length)^2,14^.

In contrast to these models of acute exposure^12–15,21^ and those focussing on the role of IL-17A^2,14^, the present model employed chronic (24 weeks) and low-level pro-inflammatory (to cigarette smoke) conditions, which can lead to emphysematous changes in wild-type mice. These lung inflammatory and structural changes were prevented by the dual *Il17a* and *Il17f* deficiency after 24 weeks, with significant attenuation of neutrophil recruitment by over 70% after 28 days of inhalation, compared to WT control animals. On the one hand, the disparities in the lung structural changes after long-term cigaretet smoke exposure, between mice deficient with *Il17a* in a literature^2,14^ and those dually deficient of *Il17a* and *Il17f* in the present study, are likely to be explained by overlapping functions between IL-17A and IL-17F^3,4,22^. On the other hand, IL-17A is associated with frequent acute exacerbations^10,23,24^, whereas IL-17F has been linked to lung cancer^25^, and asthma^26,27^ suggesting that IL-17A and F may have multiple, but indipendent roles in airway pathogenesis.

Regarding the overlapping function of IL-17A and F, previous studies showed that both IL-17A and IL-17F share the activation of p38 MAP kinase signalling, leading to the migration of both B cells and neutrophils, as well as production of chemokines, such as CCL-19 and CXCL-13^28,29^. Furthermore, IL-17 synergises with various inflammatory pathways mediated by NF-κB, cFOS, STAT-1 and Ras-Raf^6^. IL-17 enhances GM-CSF and CXCL-8 release in human cells in vitro^30–34^ and in mice in vivo^35^. Likewise, KC expression, as well as the subsequent morphometric changes, were upregulated and extended in our mouse model in the presence of IL-17A/F, together with increasing trend of other cytokines, such as TNF-α at day 8^17,18^, whereas these responses are markedly abrogated in the absence of IL-17A and F, especially after chronic exposure.

The recruited neutrophils after cigarette smoke exposure were associated with increased MMP-9 levels in BAL fluid in *Il10* deficient mice^17^, suggesting that cigarette smoke exposure induced pulmonary neutrophil recruitment via enhancing the expression of chemoattractants and that recruited neutrophils resulted in the elevation of *Mmp9* expression in the lung tissue^36^, which in turn destroyed elastin fibres in the extracellular matrix of the alveoli, resulting in enlarged alveolar airspaces. According to the present study, *Il17a* and *Il17f* dual deficiency inhibited these processes by blocking neutrophil chemoattractants and thus neutrophil recruitment. This may explain the negative result of a clinical trial of antibody against IL-17RA in severe asthma, since it had no inclusion criteria for neutrophilic inflammation^37^. Both studies using mice deficient with *il17ra*^14,38^, and a clinical study showing the no efficacy of blocking IL-17A to COPD patients^39^ strongly indicate that inhibition of both IL-17A and IL-17F, or alternative inhibition of their receptor, IL-17RA, may be needed for effective suppression of neutrophilic airway inflammation.

### Limitations

The present study has some limitations. First, since mouse models of COPD do not completely mimic the human disease, the pro-inflammatory effect of *Il17a* and *Il17f* may not occur in human COPD. However, several studies have demonstrated that the overlapping mechanisms between IL-17A and IL-17F are shared both in humans and mice^2,4,22^. Second, the lung morphometric measurements in the air-exposed mice with dual deletion of *Il17a* and *Il17f* appeared to range between those of air-exposed and cigarette smoke exposed wild type mice, suggesting some additional mechanism is involved. Third, protein levels and mRNA expression may dissociate, and the measuring protein level of these chemoattractants may indicate possible post-transcriptional effects of IL-17 deficiency on KC and MIP-2. However, a previously published report showed that cigarette smoke exposure in mice increases protein levels of KC and MIP-2 in BAL fluid in parallel to the upregulation of mRNA levels^40^. In addition, even low-level but chronic gene expression may play a crucial role in the development of chronic inflammatory disorders, in which the mediator levels in BAL fluid may be unmeasurable because of dilution, so that mRNA levels are more reliable measurements. Forth, the present study did not include any physiologic assessments, and is restricted to the histologic analysis of the lungs to show alveolar wall destruction, but previous studies have shown that mice exposed to cigarette smoke using the same model show exercise-induced impairment in oxygenation^19^. In addition, elevation in IL-17A levels and decrease in IL-10 levels are described in COPD patients^9,41^, and IL-17/IL-10 ratio was associated with disease severity, as measured by % predicted FEV_1_ values in COPD patients^41^, suggesting that IL-17 deficiency is likely to be associated with impairment in physiologic measures. Fifth, comparison between the effect of dual deficiency of *il17a* and *il17f* and single deficiency of *il17a* in a literature^2,14^ showed that the former was exclusively associated with restored airspace enlargement after cigarette smoke exposure for 24 weeks. However, this difference may possiblly be accounted for by *il17f* deficiency, which will be addressed by the comparison between mice dually deficient with *il17a* and *il17f* and those simply with *il17f* deficiency. Finally, other MMPs, such as MMP-2 and MMP-12, have also been shown to be involved in the pathogenesis of COPD^42,43^. However, the present study focused exclusively on the association between IL-17 and MMP-9, because they are separately described as major players in COPD development^19,41^, and their association is well described in cellular experiments^36^.

### Concluding remarks

In conclusion, *Il17a* and *Il17f* dual deficient mice showed reduced neutrophilic inflammation in responses to short-term cigarette exposure and the complete protection from airspace enlargement induced by long-term exposure, which was not found in *il17a* deficient mice. This suggests that both IL-17A and F play an interactive role in the development of emphysema, so that these cytokines or their common receptors need a total block to provide the maximum benefit in COPD and severe neutrophilic asthma.

## Methods

### Animals

C57BL/6J (B6) male mice (wild type) were purchased from CLEA Japan Inc (Tokyo, Japan) and mice deficient in both *Il17a* and *Il17f* of B6 genetic background were kind gift from Professor Y Iwakura (Tokyo University of Science). Mice dual deficient in *Il17a* and *Il17f* were obtained from pairs of dual deficient mice. The parental pairs were genotyped and confirmed to be dual deficient, using the primer sets for genomic genes for IL-17A and F (shown in Table S1)^4,44^. Animals were acclimatized for a week before the study. Male mice were exclusively used in this study, because female mice have three-day long estrous cycle of MIP-2 levels and neutrophilic migration to vagina^45,46^, which were avoided in our laboratory^17–20^. All experimental animals were housed in a specific pathogen-free unit at the animal facility with sterile bedding, food, and water. The number of the mice was decided according to the previously published study from other researchers, who used 4 to 10 mice in a group^13^. All the methods performed were reviewed and approved by the Experimental Animal Ethics Committee at Kyorin University (No.32, 2009), in accordance with the guideline by the Ministry of Education, Culture, Sports, Science and Technology, Japan^47^, and furthermore, with ARRIVE guidelines^48^.

### Exposure of mice to cigarette smoke

Two experiments were conducted, one investigating the time-course of inflammatory cellular response and the other measuring structural changes after cigarette smoke exposure in *Il17a/f* deficient mice compared to their wild-type counterparts, using the method previously described with some modifications^17–20^. In the former experiments, WT mice, aged 6–10 weeks old were exposed to cigarette smoke daily for 3, 5, 8, 12 and 28 days in a consecutive way by placing mice in a nose-only cigarette smoke inhalation experiment system for small animals (INH06- CIGR02A; MIPS, Osaka, Japan). Mice were exposed to compressed air with 6% cigarette smoke for 1 h/day, which were generated from commercially available, unfiltered Peace cigarettes (20 cigarettes/day, with 28 mg tar and 2.3 mg nicotine per cigarette; Japan Tobacco Inc., Tokyo, Japan).

In the long-term exposure studies, *Il17a/f*-deficient mice (n = 10 per group), aged 6–10 week old, and age-matched wild-type mice (n = 5–10 per group) were exposed to cigarette smoke daily or to continuous environmental air, starting at day 0, for 0, 5, 8, 12 and 28 days to make cellularity assessment at day 0,5,8, 12 and 28, respectively, for 8 days to measure mRNA levels, as well as histologic analyses, and for 24 week to make morphometric analyses on airspace enlargement (Figure S1).

Exposed and control mice were sacrificed 24 h after the last exposure, and bronchoalveolar lavage (BAL) was performed and lung tissue was sampled immediately upon sacrifice. BAL fluid was collected as described previously^17–20^. Briefly, mice were anesthetized and sacrificed with an overdose of pentobarbital (100 mg/kg i.p.; Schering-Plough, Kenilworth, NJ) 24 h after the last exposure to cigarette smoke. One mL aliquot of Hanks’ balanced salt solution (HBSS, Thermo Fisher Scientific, MA, USA) was instilled via the tracheal cannula and BAL fluid was recovered by gentle manual aspiration and repeated for 3 times. In each animal, approximately 95% of the BAL fluid was recovered. BAL fluid was centrifuged at 300×*g* for 5 min, and cell pellets were resuspended in 0.25 ml of HBSS. Total cells were counted in a Buerker chamber. Following staining with Diff Quick solution (International Reagents, Kobe, Japan), differential cell number (macrophages, neutrophils, lymphocytes) were counted on cytocentrifuged preparations (Auto smear CF-120; Sakura Finetek, Tokyo, Japan).

### Histology and immunohistochemistry

Histological and immunohistochemical analyses were conducted, according to a previously published study^17^. Briefly, anti-mouse MMP-9 polyclonal antibody (Abcam, Cambridge, UK) was used for immunohistochemical analysis at a dilution of 1:1000. A heat-induced epitope retrieval technique was used for immunohistochemical analysis. Three micron thick sections were obtained from the paraffin blocks. Immunohistochemical staining was performed using an automated immunostainer (Ventana Inc, Tuscot, AZ). All sections used for immunohistochemistry were counterstained with hematoxylin. Tissue sections processed without the addition of primary antibody were used for the negative control. Four slides were evaluated from each mouse; 10 independent high-power fields were randomly photographed, and the number of positively stained cells was counted and evaluated by a blinded observer.

### Quantitative reverse transcriptional polymerase chain reaction (qRT-PCR)

Quantitative reverse transcriptional polymerase chain reaction (qRT-PCR) were conducted, according to a previously published study^17,18^. Briefly, following recovery of BAL fluid samples, whole lungs were rapidly excised en bloc and frozen in liquid nitrogen. Gene expression levels in samples of lung tissue were determined using qRT-PCR. Total RNAwas extracted from whole lung samples using an RNeasy kit (Qiagen, Hamburg, Germany), cDNA was prepared using the Im Prom II reverse transcription system (Promega, Madison, WI, USA) for reverse transcription, and all procedures were conducted according to the manufacturer’s instructions. Murine *Kc*, *Gmcsf*, *Mmp9*, and Glyceraldehyde-3-phosphate dehydrogenase (*Gapdh*) were measured using real-time PCR, which was performed with reaction mixtures containing SYBRPremix Ex Taq (Takara, Tokyo, Japan) on a 7500 real-time PCRsystem (Applied Biosystems, Foster City, CA). Target cDNAs were amplified using the primers listed in Table 1. The relative amount of each target gene transcript was determined by dividing the calculated value of each target gene by the calculated value of *Gapdh*, as an internal control for each sample.

**Table 1:** Sequences of the PCR primers used in this study.

Gene	Forward primer sequence	Reverse primer sequence
*Gapdh*	5′-ACTCCACTCACGGCAAATTCAACGG-3′	5′-AGGGGCGGAGATGATGACCC-3′
*CXCL-1 (Kc)*	5′-GCTGGGATTCACCTCAAGAA-3′	5′-TGGGGACACCTTTTAGCATC-3′
*Gmcsf*	5′-AGATATTCGAGCAGGGTCTAC-3′	5′-GGGATATCAGTCAGAAAGGTT-3′
*Mip2*	5′-AGTGAACTGCGCTGTCAATG-3′	5′-TTCAGGGTCAAGGCAAACTT-3′
*Mmp9*	5′-TTGAGTCCGGCAGACAATCCTTGC-3′	5′-CCTTATCCACGCGAATGACGCTCT-3′

### Image analysis and lung morphometric analysis

Airspace enlargement was quantified using Image J software (National Institute of Health, https://imagej.nih.gov/ij/), according to the method previously published with some modification^49–51^. Briefly, a line grid was used to count the number of lines that ended on or intercepted alveolar tissue and automatically analyzed by the software, and mean linear intercepts (MLI) were calculated from the data^49–51^ Alveolar walls were also detected automatically, and the total area were calculated, in addition to the count of alveoli. If each air space becomes larger, MLI and air space area are both increased, while alveolar counts are lower. For the assess of airspace enlargement, three indices were employed: mean linear intercept (MLI), area of each alveolar airspace, and the number of alveoli per unit area; theoretically, if airspace is enlarged, MLI increases, the area of each alveolar airspace becomes larger, and the number of alveoli per unit area decreases.

### Statistical analysis

Statistical analyses of the data were made, using the PRISM version 4, (GraphPad Software, San Diego, CA, USA) and Statistical Package for Social Science (SPSS) version 17.0 for Windows (SPSS Inc., Chicago, IL, USA). Values are expressed as mean ± standard error (SE). Kruskal–Wallis tests, in conjunction with post-hoc Dunn’s tests, were performed for comparisons between four groups, if the former test revealed statistically significant. Mann–Whitney U-tests were performed in order to make an estimation of the comparison between two groups. Two-sided P-values < 0.05 were considered to be statistically significant.

### Statement of ethics

The experiments and protocols were approved by the Experimental Animal Ethics Committee at Kyorin University.

## Supplementary Information

Supplementary Information.

## Abbreviations

BAL: Bronchoalveolar lavage
COPD: Chronic obstructive pulmonary disease
JAK: Janus activated kinase
KC: Keratinocyte-derived chemokine
STAT: Signal transduction activated transcription factor
