# Supplementary material for: Dual interleukin-17A/F deficiency protects against acute and chronic response to cigarette smoke exposure in mice
Source: Sci Rep. 2021 Jun 1;11:11508. doi: 10.1038/s41598-021-90853-9 (PMC8169846; doi:10.1038/s41598-021-90853-9)
Supplement: Supplementary file 1 — Supplementary Information. [file 41598_2021_90853_MOESM1_ESM.pdf]

# Dual Interleukin-17A/F Deficiency Protects against Acute and Chronic Response to Cigarette Smoke Exposure in Mice

Hiroo Wada[a,b,k], Masuo Nakamura[b,j,k], Shin-Ichi Inoue[c], Akihiko Kudo[d], Tomoko Hanawa[e], Yoichiro Iwakura[f], Fumie Kobayashi[g], Hiroshi Kamma[h], Shigeru Kamiya[e], Kazuhiro Ito[i], Peter J Barnes[i], and Hajime Takizawa[b]

**Affiliations:**

[a]Department of Public Health, Graduate School of Medicine, Juntendo University, 2-1-1 Hongo, Bunkyo, Tokyo 113-8421, JAPAN.

[b]Departments of Respiratory Medicine, Kyorin University School of Medicine, Shinkawa, Mitaka, Tokyo 181-8611, JAPAN

[c]Division of Immunology, Department of Molecular Microbiology and Immunology, Graduate School of Biomedical Sciences, Nagasaki University, 1-12-4, Sakamoto, Nagasaki, Nagasaki 852-8523 JAPAN

[d]Department of Microscopic Anatomy, Kyorin University School of Medicine, Shinkawa, Mitaka, Tokyo 181-8611, JAPAN

[e]Department of Infectious Diseases, Kyorin University School of Medicine, Shinkawa, Mitaka, Tokyo 181-8611, JAPAN

[f]Division of Animal Experimental Immunology, Research Institute for Biological Sciences, Tokyo University of Science, 2641 Yamazaki, Noda, Chiba 278-8510, JAPAN

[g]Department of Environmental Science, School of Life and Environmental Science, Azabu University, Chuo-ku, Sagamihara City, Kanagawa 252-5201, JAPAN

[h]Department of Pathology, Kyorin University School of Medicine, Shinkawa, Mitaka, Tokyo 181-8611, JAPAN

[i]Airway Disease Section, National Heart and Lung Institute, Imperial College London

[j]Present address is Nakamura Clinic, 2-44-15 Kami-ishiwara, Chofu, Tokyo, JAPAN

[k]Those authors equally contributed to the work.

# Supplementary Figure S1

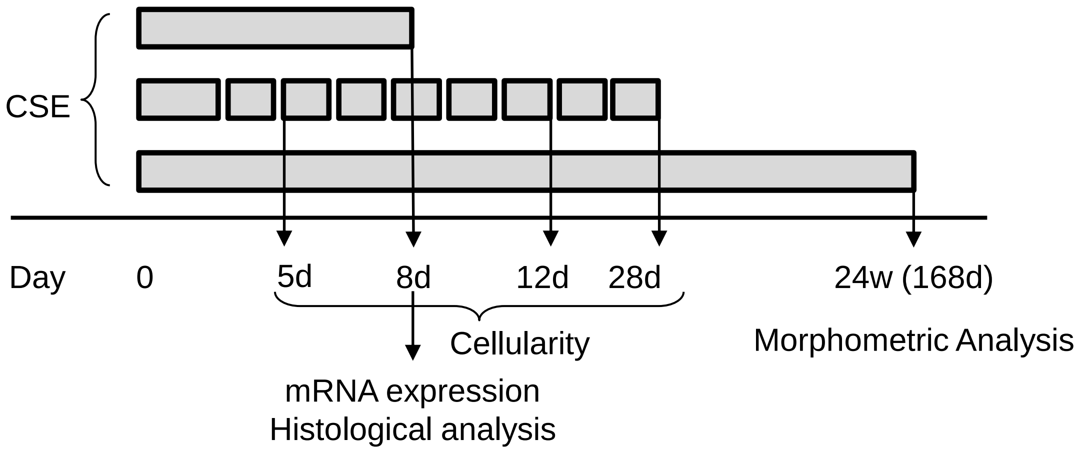

# Supplementary Figure Legends

## Supplementary Figure S1

Sampling procedures were shown.  Dual *Il17a/f* -deficient mice and age-matched wild-type mice were exposed to cigarette smoke daily or to continuous environmental air, starting at day 0, for 0, 5, 8, 12 and 28 days and 24 weeks. Cellularity assessment was made at day 0,5,8, 12 and 28.  Measurement of mRNA levels, as well as histologic analyses, were made at day 8.   Morphometric analyses on airspace enlargement were made at 24 weeks.

# Supplementary Table S1

Supplementary Table S1. Sequences of the PCR primers to detect wild type and mutant alleles

| Gene | Primer name | primer sequence |
|---|---|---|
| Il17a | Primer 1 | 5′-ACTCTTCATCCACCTCACACGA-3′ |
| | Primer 2 | 5′-GCCATGATATAGACGTTGTGGC-3′ |
| | Primer 3 | 5′-CAGCATCAGAGACTAGAAGGGA-3′ |
| Il17f | Primer 4 | 5’- TGG TAC TGC ATC AAA GTG ACA GTC -3’ |
| | Primer 5 | 5’- AAG GGT TCA GAG TCT GCG CTG CTC -3’ |
| | Primer 6 | 5’- GGA AGA TAG CAG GCA TGC TGG -3’. |
